# The uniform-score gene set analysis for identifying common pathways associated with different diabetes traits

**DOI:** 10.1186/s12864-015-1515-3

**Published:** 2015-04-23

**Authors:** Hao Mei, Lianna Li, Shijian Liu, Fan Jiang, Michael Griswold, Thomas Mosley

**Affiliations:** Center of Biostatistics & Bioinformatics, University of Mississippi Medical Center, Jackson, MS USA; Shanghai Children’s Medical Center, Shanghai Jiao Tong University School of Medicine, Shanghai, China; Department of Biology, Tougaloo College, Jackson, MS USA; Department of Neurology, University of Mississippi Medical Center, Jackson, MS USA

**Keywords:** GWAS, Pathway, Diabetes

## Abstract

**Background:**

Genetic heritability and expression study have shown that different diabetes traits have common genetic components and pathways. A computationally efficient pathway analysis of GWAS results will benefit post-GWAS study of SNP associations and identification of common genetic pathways from diabetes GWAS can help to improve understanding of the disease pathogenesis.

**Results:**

We proposed a uniform-score gene-set analysis (USGSA) with implemented package to unify different gene measures by a uniform score for identifying pathways from GWAS data, and use a pre-generated permutation distribution table to quickly obtain multiple-testing adjusted p-value. Simulation studies of uniform score for four gene measures (*minP*, *2ndP*, *simP* and *fishP*) have shown that USGSA has strictly controlled family-wise error rate. The power depends on types of gene measure. USGSA with a two-stage study strategy was applied to identify common pathways associated with diabetes traits based on public dbGaP GWAS results. The study identified 7 gene sets that contain binding motifs at promoter region of component genes for 5 transcription factors (TFs) of FOXO4, TCF3, NFAT, VSX1 and POU2F1, and 1 microRNA of mir-218. These gene sets include 25 common genes that are among top 5% of the gene associations over genome for all GWAS. Previous evidences showed that nearly all of these genes are mainly expressed in the brain.

**Conclusions:**

USGSA is a computationally efficient approach for pathway analysis of GWAS data with promoted interpretability and comparability. The pathway analysis suggested that different diabetes traits share common pathways and component genes are potentially regulated by common TFs and microRNA. The result also indicated that the central nervous system has a critical role in diabetes pathogenesis. The findings will be important in formulating novel hypotheses for guiding follow-up studies.

**Electronic supplementary material:**

The online version of this article (doi:10.1186/s12864-015-1515-3) contains supplementary material, which is available to authorized users.

## Background

Genome-wide association studies (GWAS) have been successful in identifying risk genetic variants for various human complex traits, and many GWAS have been deposited into dbGaP with genome results publicly available [1]. Effective analyses of these existing GWAS results will benefit post-GWAS study of SNP associations and improve genetic analyses of complex diseases.

The majority of existing GWAS are based on single-SNP association tests; however, single-SNP GWAS have some critical limitations. A major concern is that most identified variants are out of the gene boundary and present only modest effect individually [2]. A large number of SNP tests require the use of stringent significance criteria (e.g. p-value ≤ 5 × 10 ^−8^), which will lead to misidentification of SNPs with weak effects. Genetic heterogeneity can result in the presence of different risk variants in a gene at different GWAS, which further decreases study power and reduces replicability. Besides, common diseases like diabetes are essentially due to the effects of multiple genes, and it is difficult to extrapolate biological processes from single-SNP findings. Gene-set analysis (GSA), in contrast, hypothesizes that a common disease is influenced by polygenic factors, and GSA aims to test for associations between curated pathways and a phenotype through SNP associations [3]. In contrast to single-SNP GWAS, GSA examines SNPs inside the gene boundary and focuses on component gene associations of a curated gene set. Pathway study alleviates GWAS limitations and can contribute to the discovery of systematic genetic regulation underlying complex diseases.

The p-value-based GSA is a common type of pathway analysis that does not require access to individual SNP genotypes; the analysis is based only on association p-values of SNPs over the genome, making the analysis broadly applicable for any GWAS with few limitations. GSA typically requires measuring gene associations from all SNPs mapped to genes. A straightforward gene measurement approach is to use the minimum p-value of SNP associations [3-6]. The second best p-value of SNP associations is also used as a gene measure to evade some spurious associations [7]. However, these measures are generally incomparable and hard to interpret. For example, the best p-value as a gene measure may be smaller than 0.05, but it is difficult to interpret the gene association without comparing to other genes; besides, the best p-value is always smaller than the second best p-value, but it does not indicate a stronger gene association than the other.

For a curated gene set, an enrichment score is often calculated from its component genes as a statistic to measure pathway association. A common score is a Kolmogorov-Smirnov-like statistic [4,8] calculated over gene measures. Other effective measures include a count of significant genes [6,9], the ratio of nominally significant (P < 0.05) to non-significant SNPs [10], max mean and re-standardization of gene measures [7]. These measures typically require a large number of permutations to obtain an association p-value, and the high computational load may impede the GSA application. Furthermore, the enrichment score is gene-measure dependent, and the permutation is study- and sample-specific; this makes results difficult to compare among different studies. The Z-statistic method is a parametric measure of pathway association [7] without requiring permutation; however, the test requires an assumption of gene-set independence. To supplement existing methods and address their potential limitations, we proposed a uniform-score GSA (USGSA) that aims to improve specificity of pathway and promote interpretability and comparability of pathway results with high computational efficiency.

Diabetes is a chronic metabolic disease of hyperglycemia resulting from defects in insulin secretion, action, or both. Its prevalence continues to increase, and is anticipated to rise to 366 million worldwide in 2030 [11]. Diabetes has two major types accompanied with varied symptoms and complications. Type I diabetes (T1D) is known as insulin-dependent diabetes and it is believed to be caused by destruction of beta cells with subsequently absolute lack of insulin. Type II diabetes (T2D) is non-insulin-dependent diabetes and it is mainly characterized by insulin resistance with subsequently relative lack of insulin and hyperglycemia, for which beta cells in contrast can still produce and secrete insulin. A pathway study can help researchers understand the genetic basis of diabetes pathogenesis and design effective strategies for alleviating the public heath burden of diabetes. Diagnostic criteria recommended by the American Diabetes Association include lab testing of hemoglobin A1c (HbA1c), fasting plasma glucose (FPG), glucose tolerance and hyperglycemia [12]. The long-term effects of diabetes also cause different complications, including diabetic nephropathy [12]. Identification of common genetic pathways underlying these symptoms and complications can provide clues to better understand the etiology, pathophysiology changes and progress of diabetes.

Genetic heritability of T1D is as high as 88% [13]. The concordance rate of type 2 diabetes (T2D) is 50–92% for monozygotic (MZ) twins, consistently greater than the rate for dizygotic (DZ) twins [14]. The complication of diabetic nephropathy presented familial clustering [15]. Twin bivariate genetic study of the Atherosclerosis Risk in Communities (ARIC) population showed that genetic heritability is 30% for fasting glucose and 39% for fasting insulin, and genetic correlation between them is 22% ~ 39% [16]. Gene expression study evidenced that T1D and T2D share common pathways which are likely related to hyperglycemia and beta-cell dysfunctions [17]. These evidences suggest strong genetic susceptibility to diabetes traits and indicates shared genetic components and pathways among diabetes traits. The current study aims to identify common pathways associated with diabetes traits by analyzing dbGaP GWAS data, and we expect that the high specificity of USGSA using an independent distribution table of pathway associations will make the findings replicable and comparable among studies.

## Results

### Simulation study of family-wise error rate and power

**Figure 1:**
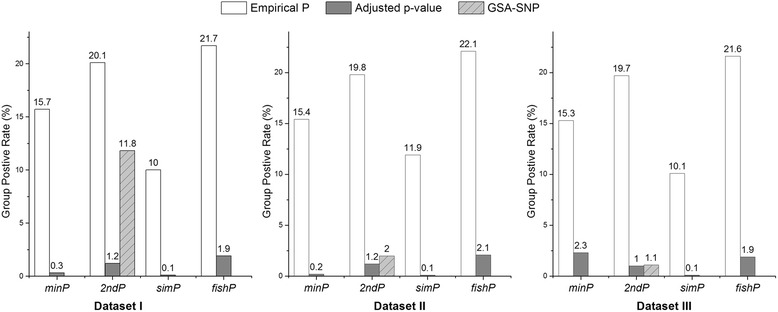
Estimate of USGSA family-wise error rate by group positive rate.

Dataset I, II and III of null pathway association were examined by USGSA with gene measures of *minP*, *2ndP*, *simP* and *fishP*. The false group positive rate (GPR) of identifying significant pathways was calculated to estimate the family-wise error rate (FWER), and results are shown in Figure 1. The false GPR based on pathway empirical p-value (*p*_*e*_) was *15.7%, 15.4%* and *15.3%* for *minP*, *20.1%, 19.8%* and *19.7%* for *2ndP*, *10%*, *11.9%* and *10.1%* for *simP*, and *21.7%*, *22.1%* and *21.6%* for *fishP* at the three datasets. The results showed that the *p*_*e*_ based on hypergeometric distribution function has inflated FWER due to multiple testing. In contrast, the false GPR based on pathway adjusted p-value (*p*_*adj*_) was *0.3%, 0.2%* and *2.3%* for *minP*, *1.2%, 1.2%* and *1.0%* for *2ndP*, *0.1%*, *0.1%* and *0.1%* for *simP*, and *1.9%*, *2.1%* and *1.9%* for *fishP* at the three datasets. The results showed that the *p*_*adj*_ based on pre-generated permutation table has well-controlled FWER. For comparison, a computationally efficient approach, GSA-SNP with corrected p-values for multiple testing, was also examined and the GRP is *11.8%* at dataset I, *2%* at dataset II, and *1.1%* at dataset III. The results demonstrate that the corrected p-values of GSA-SNP may also have increased Type I error.

**Figure 2:**
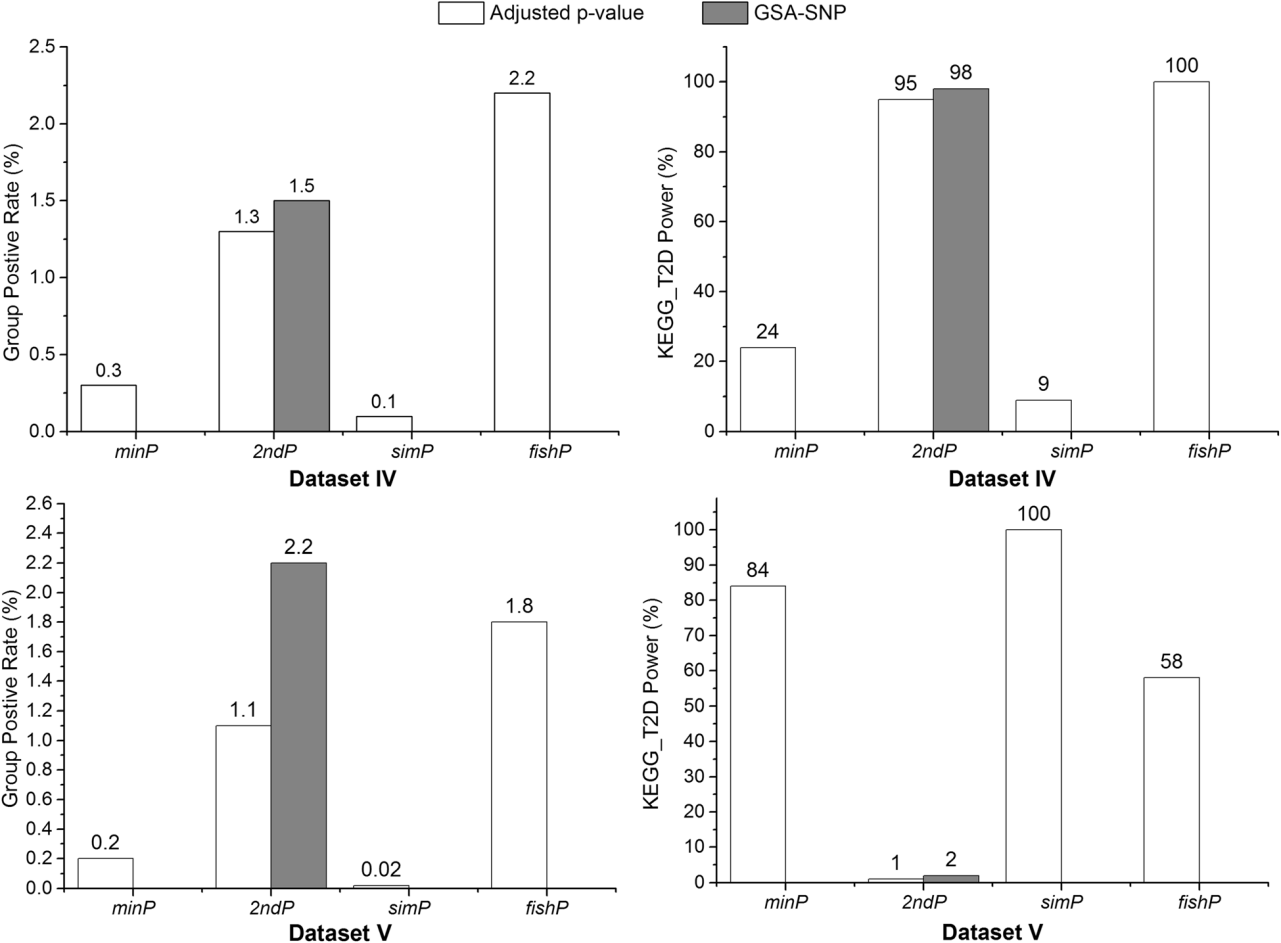
Estimate of USGSA power by group positive rate.

Dataset IV and V were examined to estimate specificity and power of USGSA and results are presented in Figure 2. Since only component genes of KEGG_T2D were simulated to contain SNP associations, majority of MSigDB gene sets had null pathway associations at both data sets and a small GPR of MSigDB gene sets indicated high specificity of USGSA. Pathway analysis by USGSA showed that the GPR based on adjusted p-value of *p*_*adj*_ is *0.3%* (*minP*), *1.3%* (*2ndP*), *0.1%* (*simP*) and *2.2%* (*fishP*) at dataset IV, and the value is *0.2%* (*minP*), *1.1%* (*2ndP*), *0.02%* (*simP*) and *1.8%* (*fishP*) at dataset V. The GPR of GSA-SNP approach based on corrected p-value is *1.5%* in dataset IV and *2.2%* in dataset V. The simulation studies showed that both USGSA and GSA-SNP have high specificity of pathway association test.

The power of identifying KEGG_T2D in Datasets IV and V were estimated based on the pathway *P*_*adj*_ of USGSA and the corrected p-values of GSA-SNP. Results are presented in Figure 2. The power is *24%* (*minP*), *95%* (*2ndP*), *9%* (*simP*) and *100%* (*fishP*) at dataset IV, and the value is *84%* (*minP*), *1%* (*2ndP*), *100%* (*simP*) and *58%* (*fishP*) at dataset V. Similar to *2ndP* measure of USGSA, the GSA-SNP has power of *98%* in dataset IV and *2%* in dataset V. The results demonstrate that analysis power depends on correct selection of a gene measure and the same gene measure may have contrary conclusion due to different characteristics of gene effects at two datasets. For example, the power of USGSA is *24%* at dataset IV but *84%* at dataset V for *minP*, and the power is *100%* at dataset IV but *58%* at dataset V for *fishP*. The GSA-SNP analysis uses the second best SNP p-value as gene measure, so it presents similar power as USGSA with *2ndP* measure.

### Identification of common pathways for diabetes traits

Top *500* gene sets were selected from USGSA pathway study of GWAS at stage I and validation analysis at stage II identified *7* common gene sets significantly associated with all studied diabetes traits. Characteristics of the gene sets were summarized in Table 1. Of the identifications, six gene sets share a transcription factor (TF)-binding motif at gene promoter region of [-2 kb, 2 kb] respectively: 1) pathway “1461” contains the motif of AACTTT, but the binding factor is not known; 2) pathway “2247” contains the binding motif of TTGTTT for FOXO4, which regulates the insulin signaling pathway through binding to insulin-response elements [18,19]; 3) pathway “2268” contains the binding motif of TGGAAA for NFAT, which is an activator in response to elevation of intracellular Ca2+, regulating insulin gene transcription by a Ca(2+)-responsive pathway [20]; 4) pathway “2240” contains the motif of CAGGTG for TCF3, which is up-regulated specifically in islets of T2D patients and is associated with Wnt signaling in diabetes pathogenesis [21]; 5) pathway “2239” contains the binding motif of TAATTA for VSX1; and 6) pathway “1551” contains the binding motif of NNGAATATKCANNNN for POU2F1. The 7^th^ pathway (pid: 2076) contains the target motif of AAGCACA for microRNA, mir-218.

**Table 1:** **Common gene sets associated with different diabetes traits**

**Pid**	**Size**	**Motif**	**Binding**	**Web**
1461	1890	AACTTT	Unknown	http://www.broadinstitute.org/gsea/msigdb/cards/AACTTT_UNKNOWN
2247	2061	TTGTTT	FOXO4	http://www.broadinstitute.org/gsea/msigdb/cards/TTGTTT_V$FOXO4_01
2268	1896	TGGAAA	NFAT	http://www.broadinstitute.org/gsea/msigdb/cards/TGGAAA_V$NFAT_Q4_01
2240	2485	CAGGTG	TCF3	http://www.broadinstitute.org/gsea/msigdb/cards/CAGGTG_V$E12_Q6
2076	398	AAGCACA	MIR-218	http://www.broadinstitute.org/gsea/msigdb/cards/AAGCACA,MIR-218
2239	810	TAATTA	VSX1	http://www.broadinstitute.org/gsea/msigdb/cards/TAATTA_V$CHX10_01
1551	214	NNGAATATKCANNNN	POU2F1	http://www.broadinstitute.org/gsea/msigdb/cards/V$OCT1_02

At the stage I, the identified gene sets of ‘1461’ (AACTTT-motif), ‘2247’ (FOXO4), ‘2268’ (NFAT), ‘2240’ (TCF3), ‘2076’ (MIR-218), ‘2239’ (VSX1) and ‘1551’ (POU2F1) have *11.1% ~ 13.2%, 9.7% ~ 10.8%, 9.4% ~ 11.9%, 8.8% ~ 10.0%, 13.3% ~ 18.8%, 11.1% ~ 14.2%* and *17.7% ~ 22.3%* of component genes with uniform score *≤ 5%* respectively (Table 2)*.* This value is significantly higher than the assumed *5%* of genes in the genome associated with diabetes traits, which has pathway association p-values (*p*_*e*_) of 3.71*10^*−7*^ ~ 3.44*10^*−28*^ (Table 2). The measures of gene set associations over GWAS of stage I showed that gene sets of ‘1461’, ‘2247’, ‘2268’, ‘2240’, ‘2076’, ‘2239’ and ‘1551’ have chi2 (rank) of *1008.84 (13)*, *626.50 (14)*, *596.97 (15)*, *415.33 (21), 474.90 (17)*, *462.18 (18)* and *499.03 (16)* respectively.

**Table 2:** **Common gene sets associated with different diabetes traits**

**GWAS\PID**	**1461**	**2247**	**2268**	**2240**	**2076**	**2239**	**1551**
	**Stage I:** ***p***_***e***_ **(sig%)**						
pha000417	2.89E-21	5.77E-11	3.34E-14	1.90E-12	9.98E-16	1.78E-07	2.29E-08
	(12.1)	(9.8)	(10.8)	(10.0)	(18.8)	(11.1)	(17.7)
pha000423	2.57E-18	2.25E-12	1.06E-19	2.19E-08	9.39E-08	1.45E-07	5.07E-09
	(11.5)	(10.1)	(11.9)	(9.0)	(13.7)	(11.1)	(18.4)
pha000427	2.82E-21	1.91E-13	1.64E-10	3.00E-09	1.52E-11	6.56E-08	2.89E-13
	(11.9)	(10.2)	(9.7)	(9.1)	(16.0)	(11.1)	(22.2)
pha000429	1.60E-23	5.14E-11	1.81E-09	8.90E-08	2.69E-11	1.07E-10	2.08E-10
	(12.4)	(9.7)	(9.6)	(8.8)	(16.0)	(12.5)	(19.7)
pha000433	5.38E-20	4.47E-15	3.19E-11	3.41E-08	1.35E-12	5.28E-08	4.27E-10
	(12.0)	(10.8)	(10.2)	(9.1)	(17.1)	(11.4)	(19.6)
pha000437	1.06E-13	8.91E-13	7.21E-11	5.99E-08	2.46E-07	1.91E-09	1.15E-09
	(10.5)	(10.2)	(10.0)	(8.9)	(13.3)	(12.0)	(19.0)
pha000447	1.08E-23	4.96E-12	9.30E-16	1.30E-08	4.90E-09	9.70E-09	6.31E-12
	(12.2)	(9.8)	(10.9)	(8.9)	(14.3)	(11.4)	(20.9)
pha000451	1.01E-15	1.15E-14	2.67E-08	1.19E-08	6.53E-10	4.79E-11	3.01E-08
	(11.1)	(10.7)	(9.4)	(9.2)	(15.4)	(12.8)	(17.7)
pha000453	3.44E-28	5.34E-15	1.10E-12	6.90E-09	1.18E-09	5.22E-15	4.56E-13
	(13.2)	(10.7)	(10.4)	(9.2)	(15.0)	(14.2)	(22.3)
pha000457	3.20E-25	7.46E-15	2.61E-14	2.58E-09	3.71E-07	3.49E-11	1.93E-11
	(12.9)	(10.7)	(10.9)	(9.4)	(13.3)	(12.9)	(20.9)
pha000461	3.68E-16	7.82E-12	1.36E-10	1.04E-8	4.46E-9	2.80E-11	7.52E-11
	(11.1)	(10.0)	(10.0)	(9.2)	(14.7)	(12.8)	(20.3)
	**Stage I: Chi2 (Rank)**						
	1008.84 (13)	626.50 (14)	596.97 (15)	415.33(21)	474.90 (17)	462.18 (18)	499.03 (16)
	**Stage II:** ***p***_***e***_ **(sig%)** ***(p***_***adj***_ **)**						
pha002839	8.3E-35	5.78E-21	3.73E-18	4.60E-22	1.61E-11	1.73E-16	1.28E-8
	(13.8)	(11.5)	(11.3)	(11.0)	(15.4)	(14.1)	(17.2)
	(<1E-4)	(<1E-4)	(<1E-4)	(<1E-4)	(<1E-4)	(<1E-4)	(4E-4)
pha002862	6.70E-20	1.41E-13	1.34E-15	2.14E-12	5.85E-13	8.35E-8	3.33E-11
	(11.4)	(10.0)	(11.3)	(9.4)	(16.0)	(10.7)	(19.2)
	(<1E-4)	(<1E-4)	(<1E-4)	(<1E-4)	(<1E-4)	(0.003)	(<1E-4)
pha002864	2.23E-32	2.11E-21	1.43E-18	8.33E-20	2.67E-10	3.95E-11	1.10E-14
	(13.3)	(11.4)	(11.3)	(10.6)	(14.6)	(12.2)	(22.2)
	(<1E-4)	(<1E-4)	(<1E-4)	(<1E-4)	(1E-4)	(<1E-4)	(<1E-4)
pha003005	6.84E-29	2.64E-15	2.72E-13	2.47E-15	1.72E-9	4.51E-11	6.41E-7
	(13.0)	(10.5)	(10.4)	(10.0)	(14.3)	(12.3)	(15.5)
	(<1E-4)	(<1E-4)	(<1E-4)	(<1E-4)	(1E-4)	(<1E-4)	(<1E-4)
pha002901	6.45E-42	9.05E-23	4.39E-17	2.47E-20	8.43E-12	6.59E-15	2.28E-11
	(14.7)	(11.8)	(11.2)	(10.8)	(15.7)	(13.6)	(19.7)
	(<1E-4)	(<1E-4)	(<1E-4)	(<1E-4)	(<1E-4)	(<1E-4)	(<1E-4)

*p*_*e*_: Pathway empirical p-value; sig%: percentage of genes in the gene set that are at the top 5% of gene association over genome; *p*_*adj*_: Pathway adjusted p-value; *Chi*2 = ∑_*i*_ − 2 x *log*((*p*_*e*_)_*i*_) is the summary of a gene set’s association over all stage I GWAS; Rank: the rank of chi2 in decreasing order.

Pathway analysis of the 7 gene sets at stage II gave consistent results as stage I. The gene sets of ‘1461’, ‘2247’, ‘2268’, ‘2240’, ‘2076’, ‘2239’ and ‘1551’ have *11.4% ~ 14.7%, 10.0% ~ 11.8%, 10.4% ~ 11.3%, 9.4% ~ 11.0%, 14.3% ~ 16.0%, 10.7% ~ 14.1%* and *15.5% ~ 22.2%* of component genes with uniform score *≤ 5%*, which corresponds to *p*_*e*_ value of 6.70*10^*−20*^ ~ 6.45*10^*−42*^, 1.41*10^*−13*^ ~ 9.05*10^*−23*^, 2.72*10^*−13*^ ~ 1.43*10^*−18*^, 2.14*10^*−12*^ ~ 4.60*10^*−22*^, 1.72*10^*−9*^ ~ 5.85*10^*−13*^, 8.35*10^*−8*^ ~ 1.73*10^*−16*^ and 6.41*10^*−7*^ ~ 1.10*10^*−14*^, respectively (Table 2)*.* These gene sets are significant over all GWAS after controlling for multiple testing and most of their adjusted p-value (*p*_*adj*_) are *<10*^*−4*^.

**Figure 3:**
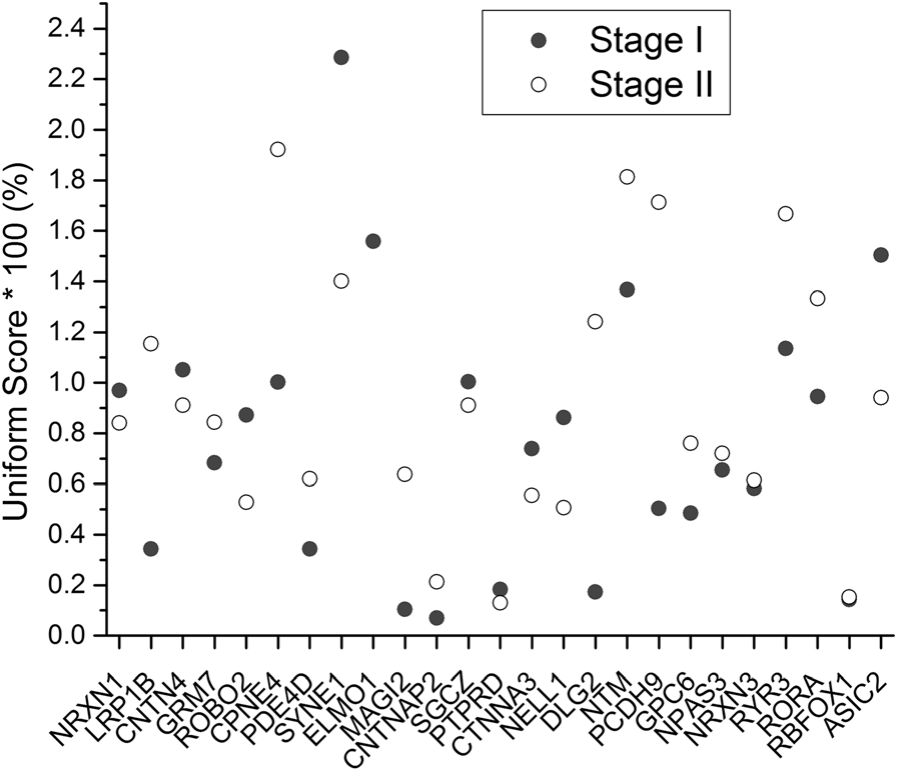
Average uniform score of significant genes for stage I and II GWAS.

The identified pathways with pids of “1461”, “2247”, “2268”, “1551”, “2239”, “2076”, and “2240” respectively contained *18, 10, 8, 4, 4, 7*, and *8* significant common genes for all GWAS in stage I and II, resulting in 25 unique common genes. These genes and their corresponding pathways are summarized in Table 3. Mean uniform scores of these genes for stage I and II are presented in Figure 3. The results demonstrate that these genes had ranges of *0.07% ~ 2.29%* at the stage I analysis and *0.13% ~ 2.74%* at the stage II analysis, indicating that these genes are among the top 3% of the gene associations with diabetes traits over genome. Detailed uniform scores are noted in Additional file 1: Table S2. Our literature and gene annotation review (Additional file 2: Table S3) showed that almost all of these genes are mainly expressed in the brain, and most are related to neurodevelopment and brain function, including schizophrenia, autism, Alzheimer's disease, impaired learning, and intellectual disability. The identified common pathways and their consistently significant genes suggest that the pathogenesis of diabetes may be attributable to microRNA and TF-mediated regulation and the central nervous system (CNS) plays important role in the regulation.

**Table 3:** **Significant genes from common gene sets associated with different diabetes traits**

**Gene**	**chr**	**Start**	**End**	**Strand**	**Pid**
NRXN1	2	50145643	51259674	-	1461/2076/2240
LRP1B	2	140988996	142889270	-	1461/2268
CNTN4	3	2140550	3099645	+	1461/2239/2240
GRM7	3	6902802	7783218	+	2247
ROBO2	3	75955845	77699115	+	2076
CPNE4	3	131252413	131759152	-	1461
PDE4D	5	58264865	59783925	-	1461/2247/2268/1551
SYNE1	6	152442819	152958534	-	1461/2240
ELMO1	7	36892511	37488895	-	1461/2268/2239/2076/2240
MAGI2	7	77646374	79083121	-	2076
CNTNAP2	7	145813453	148118090	+	1461/2268
SGCZ	8	13947373	15095792	-	2247/2076
PTPRD	9	8314246	10612723	-	2247
CTNNA3	10	67672276	69455949	-	1461/2247
NELL1	11	20691117	21597232	+	1461
DLG2	11	83166055	85338314	-	1461/2247/2268/1551/2076/2240
NTM	11	131240371	132206716	+	1461
PCDH9	13	66876966	67804468	-	1461
GPC6	13	93879078	95060274	+	1461
NPAS3	14	33404115	34273382	+	1461/2247/1551
NRXN3	14	78636716	80334633	+	1551/2076/2240
RYR3	15	33603177	34158304	+	1461/2240
RORA	15	60780483	61521502	-	1461/2247/2268/2239
RBFOX1	16	5289469	7763342	+	1461/2247/2268/2239
ASIC2	17	31340105	32483825	-	2247/2268/2240

## Discussion

We proposed the USGSA method with implemented *R* package of *snpGeneSets* to provide a convenient and fast tool for study of pathway association from GWAS data. The USGSA applies the uniform score to unify four different gene measures of *minP*, *2ndP*, *simP* and *fishP*, and measures pathway association by hypergeometric test. The pathway analysis by USGSA is based on test of MSigDB gene sets. The MSigDB annotates *10,722* genes sets with *32,364* genes, and the number is much smaller than the number of GWAS SNPs. Therefore, the pathway analysis will alleviate the burden of multiple-test adjusting and improve testing power. Application of USGSA successfully identified 7 significant gene sets associated with all studied diabetes traits, indicating common genetic regulations shared among different traits.

Four gene measures of USGSA are proposed to summarize gene effects with different characteristics: the *minP* and the *2ndP* measure gene effects based on a single-SNP association; while the *simP* and the *fishP* assess gene effects based on multiple SNP associations with accounting for the number of GWAS SNPs in a gene. USGSA applies a uniform score to unify these gene measures for comparability with the same interpretability. The score ranges from 0 to 1, and it is explained as top percentage of the gene associations over genome. The USGSA calculates empirical *p*_*e*_ of pathway association from gene measures based on hypergeometric distribution, which accounts for both the number of significant genes and the size of the pathway. The pathway adjusted p-value (*P*_*adj*_) can be calculated by permutation test to account for pathway dependence and multiple testing, and an independent pre-generated permutation table is directly used to facilitate the calculation.

USGSA gene measures can better facilitate replication studies of a gene effect that may have inconsistent SNP associations from different GWAS due to genetic heterogeneity. USGSA can also help to identify a significant pathway shared by different traits which however may be activated through different mechanisms. For example, expression study showed that T1D and T2D likely share a common pathway which however has different regulated genes (e.g. MYC) [17]. For this study, the gene set of FOXO4 (pid: ‘2247’) has total ~*2,000* genes, and contained *131 ~ 149* and *167 ~ 214* genes with uniform scores ≤ 0.05 in stage I and stage II, respectively, which are about *9.7% ~ 11.8%* of component genes among top 5% of the gene associations over genome; the gene set had only *10* significant genes over all studies. These results indicate that multiple diabetes traits may be influenced by a common pathway activated through different genes.

The USGSA does not require access to individual-level SNP genotypes and the analysis is based on GWAS p-value only. For existing pathway analysis of GWAS p-values, ALIGATOR preselects a p-value criterion to define a list of significantly associated SNPs [9]; the i-GSEA4GWAS [4], GeSBAP [5] and gamGWAS [6] all select the best p-value of SNP associations to measure gene effects; and the GSA-SNP enables selection of the k^th^ (k = 1, 2, 3, 4, or 5) best p-values as the gene measure [7]. Compared to these analyses, the USGSA provides broader measures to summarize gene effects with different characteristics as described above. The existing pathway analysis, e.g. ALIGATOR [9], generally requires selection of a p-value cut-off to identify significant SNPs and genes for pathway test. The selection may be arbitrary and study-dependent, and the results may not be comparable between different studies. In contrast, USGSA selects a uniform-score cut-point (*0 ≤ α*_*G*_ *≤ 1*) for all four gene measures, which is hypothesized as *α*_*G*_ proportion of genes associated with the study trait. Significant genes identified from different GWAS data by different USGSA gene measure will have the same interpretation, explained as top 100*α_*G*_% of the gene associations over genome. Comparing with many existing pathway analyses that rely on a time-consuming permutation to adjust for complex genetic structures, the USGSA implements the adjustment by corresponding gene measures, hypergeometric test and permutation test. In addition, benefiting from the random uniform distribution of uniform score, the USGSA provides a pre-generated permutation distribution table, which facilitates a computationally efficient calculation of pathway adjusted p-value (*P*_*adj*_) for different gene measures.

The USGSA calculates both empirical (*p*_*e*_) and adjusted (*P*_*adj*_) p-values to measure pathway association. Due to multiple testing issue, the *p*_*e*_ can result in high FWER (Figure 1). The *P*_*adj*_ was shown to have well-controlled FWER and high specificity for all four gene measures (GPR ≤ 2.3%), especially for the *simP* measure (Figures 1 and 2). GSA-SNP analysis implements a computationally efficient measure of pathway association from GWAS SNP p-values by Z statistic. However, the measure assumes independence of gene sets and the test may result in inflated FWER which is evidenced in simulation study of dataset I (GRP = 11.8%). By comparison, the power is *0.1% ~ 1.9%* for 4 gene measures of USGSA with *P*_*adj*_ as significance indicator (Figure 1). Therefore, the permutation-based *P*_*adj*_ not only adjusts for multiple testing but also alleviates potential issues due to complex genetic structure.

The power of USGSA based on *P*_*adj*_ depends on selected gene measures, and an inappropriate measure can result in a contrary conclusion. For dataset IV, component genes of KEGG_T2D pathway are simulated to have multiple SNP associations each, and the gene measures of *2ndP* and *fishP* have extremely high power of *98%* and *100%*, whereas the gene measures of *minP* and *simP* have low power of *24%* and *9%* respectively. For dataset V, *11* component genes of KEGG_T2D pathway are simulated to have an extremely strong SNP association each, and the gene measures of *minP* and *simP* presented high power of *84%* and *100%*, whereas *2ndP* and *fishP* showed low power of *1%* and *58%* respectively. Therefore, different gene measures have their adaptive tests of pathway associations: *2ndP* and *fishP* are more fitting for a gene containing multiple SNP effects, while *minP* and *simP* are more suitable for a gene having extreme SNP effects. The results also indicate that the *fishP* is tolerant to gene effects characterized with different types of SNP associations.

**Figure 4:**
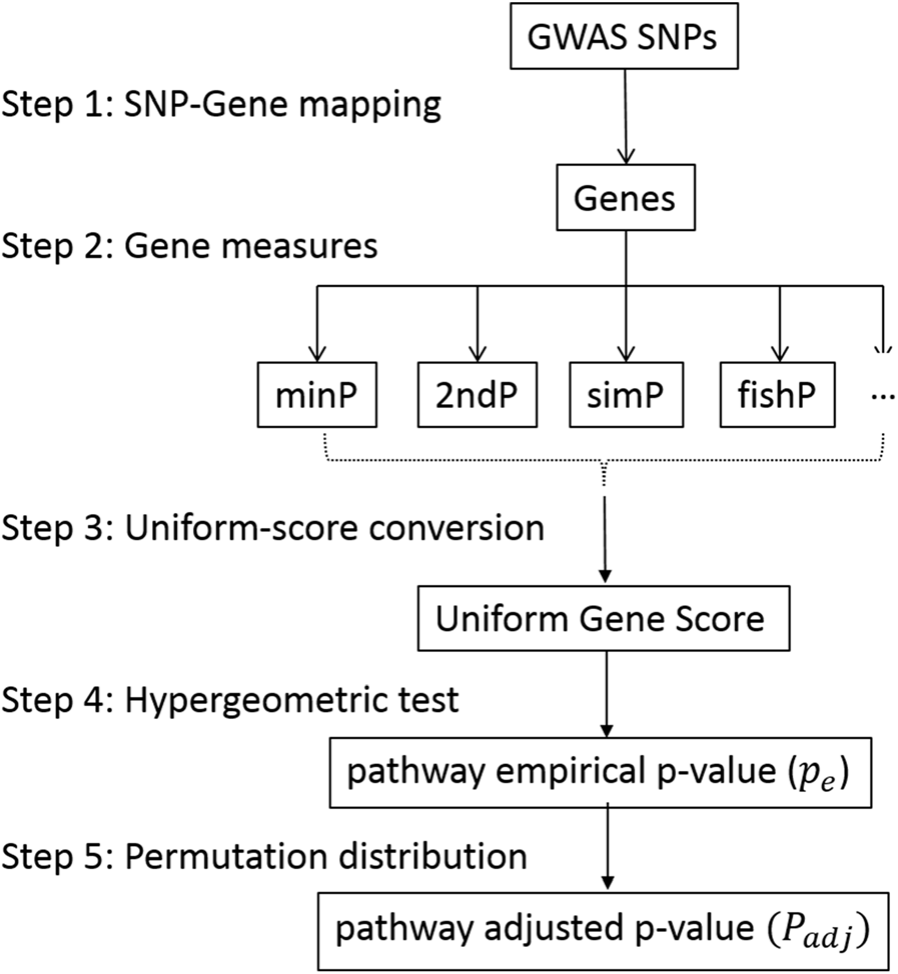
Implementation of USGSA for pathway association test.

USGSA was successfully applied to identify common pathways associated with different diabetes traits. GWAS SNP associations of diabetes traits were identified from the dbGaP and classified using *11* FHS GWAS in stage I and *5* non-FHS GWAS in stage II. GWAS analyses of the FHS samples are dependent, leading to the possibility that SNP associations are potentially correlated among different GWAS and the identified pathways in stage I may be false-positive. Therefore, we analyzed the non-FHS GWAS, based on independent samples, to validate the candidate gene sets through stage II. Although the FHS and non-FHS GWAS are respectively based on lower- and higher-resolution genotyping from different platforms, SNP-Gene mapping of USGSA (Figure 4) applies the recent genome build and makes a consistent map of SNPs and genes to perform comparable pathway studies across heterogeneous GWAS.

USGSA with the *fishP* measure successfully identified 7 common gene sets associated with diabetes traits. Component genes of these gene sets have significantly higher probability of association with diabetes traits among the top 5% of genes than a random gene. These component genes have common binding motifs in their promoter regions of [-2kb, 2kb] around transcription start sites. The motifs include targets for 5 TFs of FOXO4, NFAT, TCF3, VSX1 and POU2F1, 1 microRNA of MIR-218 and one unknown binding factor. There are 25 common component genes with uniform score ≤ 0.05 over all GWAS (Table 3). These genes and their binding factors suggest potential regulatory genetic mechanisms underlying diabetes pathogenesis. For example, CNTN4 belongs to the gene sets of VSX1 (pid: ‘2239’) and TCF3 (pid: ‘2240’). Association tests and mouse experiments have indicated that CNTN4 is an obesity–insulin targeted gene [22]. However, the gene function related to diabetes pathogenesis remains unclear. TCF3 is highly expressed in islets of T2D patients and is associated with Wnt signaling in diabetes pathogenesis [21]; and VSX1, expressed in ocular tissues, is associated with eye diseases [23]. Therefore, our findings and existing published evidences can lead to the hypothesis that mutations in the CNTN4 gene modify its binding with TCF3 in the pancreas and VSX1 in the brain and activate related genetic regulations for diabetes and its complications. NPAS3 is the consistent significant gene of all GWAS with target motifs for TFs of FOXO4 (pid: ‘2247’) and POU2F1 (pid: ‘1551’). FOXO4 [19] and POU2F1 [24] have been shown to regulate insulin signaling and glucocorticoid expression, respectively. Therefore, the component gene of NPAS3 and TFs of FOXO4 and POU2F1 can form another hypothesis of potential genetic pathways related to diabetes pathogenesis.

Our literature and gene annotation review showed that nearly all of the 25 common genes are highly expressed in the brain, and most of them are known as susceptibility genes for neural development and neurological disorders (Additional file 2: Table S3). These findings suggest that the CNS may have a critical role in diabetes pathogenesis. This conclusion is supported by previous studies of NPAS3, where gene mutations are related to the aerobiology of psychiatric illness [25,26] and the gene can induce susceptibility to diabetes for psychiatric patients [27]. Therefore, although pathway analysis at this study cannot provide direct evidences of regulatory mechanisms, the findings can help to form new hypotheses and initiate follow-up studies to ascertain pathogenetic changes of diabetes progress.

SNP associations for the analysis were retrieved from public GWAS results stored in the dbGaP. However, the number of available GWAS for diabetes traits is limited and the genotyping has low resolution (≤500K SNPs), especially for the FHS GWAS, which may cause missed identifications of some important gene and pathway associations. Therefore, more GWAS of diabetes traits are required to improve the power and replicate the findings in future studies. In addition, the gene set analysis depends on the selection of uniform-score cut-point *α*_*G*_, which is *5%* for this study. The MSigDB database includes *32,364* genes from *10,722* genes sets and this selection assumes ~*1,620* genes of them associated with every diabetes trait. Pathway analysis of diabetes traits showed that these genes are significantly enriched in the seven identified gene sets. A smaller/larger *α*_*G*_ will cause a decreased/increased number of target genes for enrichment test, which consequently affects identification of significant pathways.

The current study aims to identify common pathways significantly associated with different diabetes traits. The findings will help to discover shared genes and genetic pathways related to different diabetes symptoms and complications. A significant common gene set is observed only if it has *P*_*adj*_ ≤ 0.05 over all GWAS in stage II, and the probability of making type I error is far less than *0.05*. The 7 significant identifications by USGSA account for 0.07% of total MSigDB gene sets. These gene sets contain 25 common genes, which are among the top 5% of gene association over genome for all GWAS and account for *<1.5%* of total hypothesized susceptibility genes in enrichment test. However, it may not be accurate for the hypothesis that 5% of genes in the genome (~*1,620* susceptibility genes) are associated with diabetes traits, and *1%* deviation of the hypothesis will result in changes of ~320 susceptibility genes for diabetes traits. Therefore, although there are strongly significant evidences that component genes from the 7 gene sets are associated with diabetes traits, these 25 common genes are only susceptibility candidate genes for diabetes pathogenesis, which requires further replications and experiment validations in the future study.

## Conclusions

In summary, we proposed the USGSA method with implemented R package of *snpGeneSets* to facilitate computationally efficient analysis of pathway association based on only association p-values of GWAS SNPs. USGSA applies a uniform score that unify 4 gene measures for summarizing different types of gene effects and uses a pre-generated distribution to directly obtain a pathway adjusted p-value. Simulation studies showed that USGSA has strictly controlled FWER and high specificity for all gene measures, but the power depends on the selected type of gene measure. USGSA makes pathway identification from different gene measures comparable and improves interpretability and replicability.

The pathway analysis of public dbGaP GWAS results identified 7 common gene sets significantly associated with all studied diabetes traits. Component genes of these gene sets are significantly enriched in the top 5% of gene associations with diabetes traits compared to random genes. The component genes have common promoter motifs for target TFs and microRNA, and 25 significant common genes were identified with high expression in the brain. The findings will help to discover pleiotropic genetic effects and formulate novel hypotheses of common genetic regulations underlying different diabetes symptoms and complications, which are important in guiding the follow-up studies.

## Methods

### The USGSA method and implementation

The USGSA method for pathway analysis takes five consecutive steps to test associations of curated gene sets with a phenotype based on association p-values of GWAS SNPs (Figure 4). This GSA method is implemented in an R package, *snpGeneSets*, and can be freely accessed at http://www.umc.edu/biostats_software/. Step 1 is the SNP-Gene mapping. The gene boundary is defined from the upstream region of the transcription start site (TSS) to the downstream region of the transcription end site (TES) in order to include a potential promoter region. Positions of GWAS SNPs and genes are identified based on NCBI dbSNP [28] and Gene [29] databases, respectively, of reference genome build 37. The mapping process assigns a SNP to a gene if the SNP falls inside the defined gene boundary.

The second step is ‘Gene measures’ for summarizing gene effect from SNP associations. There are four gene measures of the best p-value (*minP*), the second best p-value (*2ndP*), the Simes’ p-value (*simP*) and the Fisher’s p-value (*fishP*). For *K* SNPs mapped to a gene with GWAS p-values (*p*_*1*_*, p*_*2*_*,…p*_*k*_), the ordered p-value is defined as *p*_*(1)*_ *≤ p*_*(2)*_ *≤ … ≤ p*_*(k)*_, where p_(1)_ = min{*p*_*1*_*, p*_*2*_*,…p*_*k*_} and p_(k)_ = max{*p*_*1*_*, p*_*2*_*,…p*_*k*_}. The four measures are calculated as:

$$ \begin{array}{l} minP={p}_{(1)},\hfill \\ {}2ndP={p}_{(2)},\hfill \\ {} simP=mi{n}_i\left\{K{p}_{(i)}/i\right\},\hfill \\ {} fishP= Pr\left(X\ge x=-2{\displaystyle {\sum}_{i=1}^K log\left({p}_i\right)}\right)=\varPsi (x)\hfill \end{array} $$

where Ψ is the chi-square distribution function with *df = 2K*. All measures take values between 0 and 1 and a smaller value indicates a stronger gene association.

Every type of gene measure is converted to a uniform score in Step 3. The uniform score of the *i*-th gene is calculated as, *U*_*i*_ = (∑_*j*_*I*(*M*_*j*_ < *M*_*i*_) + 0.5 · ∑_*j*_*I*(*M*_*j*_ = *M*_*i*_))/*L*, where *M*_*i*_ is gene measure of the *i*-th gene and *L* is the total number of genes. The *U*_*i*_ estimates the percentage of genes with stronger associations than the *i*-th gene in the genome. The cumulative distribution of uniform score approximately converges to *Pr(U ≤ u) ≈ u* for a large *N*. When all genes are randomly associated with a phenotype, their uniform scores will have an approximately random uniform distribution, i.e. *U ϵ (0,1)*.

Curated gene sets are obtained from the MSigDB [30] database, which integrates heterogeneous annotations from the Kyoto Encyclopedia of Genes and Genomes (KEGG) [31], the Reactome [32], the Gene Ontology [33] and the Biocarta [34]. Based on the sources and characteristics, USGSA classifies all gene sets into 20 types (Additional file 3: Table S1) and assigns a unique pathway id (pid) for every gene set. Pathway associations are measured by hypergeometric test for every curated gene set during Step 4. A pathway empirical p-value (*p*_*e*_) of a gene set *Ω* is obtained from hypergeometric distribution and calculated as:

$$ {p}_e=1-{\displaystyle {\sum}_{i=0}^K\left(\begin{array}{c}\hfill S\hfill \\ {}\hfill i\hfill \end{array}\right)}\left(\begin{array}{c}\hfill L-S\hfill \\ {}\hfill l-i\hfill \end{array}\right)/\left(\begin{array}{c}\hfill L\hfill \\ {}\hfill l\hfill \end{array}\right), $$

where *L* is the number of GWAS SNP-mapped genes (*G*_*i*_*: 1 ≤ i ≤ L*); $l={\displaystyle {\sum}_{i=1}^LI}\left({U}_i\le {\alpha}_G\right)$ defines the number of significant genes with uniform score ≤ *α*_*G*_; $S={\displaystyle {\sum}_{i=1}^LI}\left({G}_i\in\ \Omega \right)$ and $K={\displaystyle {\sum}_{i=1}^LI}\left({G}_i\in\ \Omega \right)I\left({U}_i\le {\alpha}_G\right)$. The test depends on the selection of parameter *α*_*G*_, which is the hypothesized percentage of GWAS SNP-mapped genes associated with the phenotype over genome.

Because different gene sets can have overlapping genes, the pathways for testing may not be mutually independent, and pathway *p*_*e*_ will not follow uniform distribution. A permutation test is therefore required to generate a distribution of *p*_*e*_ and identify a pathway adjusted p-value *P*_*adj*_ with control for multiple testing and pathway dependence. Since the calculation of pathway *p*_*e*_ is dependent only on a uniform score of a gene, a sample- and study-independent permutation distribution table of *p*_*e*_ is therefore generated based on *10,000* randomly simulated datasets of uniform scores for all GWAS-SNP mapped genes in a uniform distribution. For a particular gene set, its pathway *P*_*adj*_ is therefore calculated as

$$ {P}_{adj}={\displaystyle {\sum}_{i=1}^{10,000}\left( \min \left\{{p}_{ij}\right\}\le {p}_e\right)}/10000, $$

where *p*_*ij*_ is the permuted pathway *p*_*e*_ for the *j*-th curated get set at *i*-th permutation data.

### Simulation study

We simulated three and two datasets respectively to evaluate the type I error and the power of USGSA for different gene measures. To mimic a real pathway analysis of GWAS SNP associations, we extracted SNPs from the T2D GWAS [35] for the simulation (n = *306,417)*. The gene boundary for the analysis was defined to include 20 kb regions both upstream and downstream of the transcription zone.

Dataset I was generated by simulating random SNP p-values in a uniform distribution between *0* and *1*, i.e. ~*U(0,1)*. The SNP p-value in Dataset II was simulated to follow a beta distribution with shape *α*_*1*_ *= α*_*2*_ *= 0.5*, i.e., ~*beta(0.5,0.5)*. The SNP p-value in Dataset III was randomly generated by permuting T2D-GWAS p-values [35]. Datasets I, II and III were analyzed to evaluate the Type I error of USGSA.

To evaluate power, we simulated a pathway association of gene set, KEGG_T2D [36], which consists of *47* genes. For Dataset IV, all SNPs mapped to KEGG_T2D genes had GWAS p-values in a beta distribution *beta(1,3)* with a mean SNP p-value of *0.25*, and all other SNPs had GWAS p-values in a uniform distribution *U(0,1)* with a mean SNP p-value of *0.50*. For Dataset V, two steps were taken to simulate *11* genes of KEGG_T2D associated with phenotype. For Step 1, SNP p-values were randomly simulated in a uniform distribution ~ *U(0,1)*. For Step 2, *11* genes were randomly selected from KEGG_T2D, and the minimum SNP p-value of every gene was randomly switched with the *11* minimum p-values of all GWAS SNPs. The number of *11* was determined through the inverse hypergeometric distribution function for pathway empirical *p*_*e*_ that corresponds to the pathway adjusted *P*_*adj*_ = 0.05 from the pre-generated permutation table.

Every dataset consisted of *100* GWAS, and the USGSA was applied to test pathway associations of all MSigDB gene sets at every GWAS. GSA-SNP analysis was also applied and results were compared with USGSA. The group positive rate (GPR) was estimated as the proportion of MSigDB gene sets with *P*_*adj*_ ≤ 0.05, and power was estimated as the probability of KEGG_T2D with *P*_*adj*_ ≤ 0.05.

### Pathway analysis of diabetes traits

USGSA was applied to identify common pathways associated with different diabetes traits, which took *fishP* as gene measure and assumed 5% of genes over genome associated with diabetes traits (i.e. *α*_*G*_ *= 0.05*). GWAS results of diabetes traits were obtained from dbGaP [1]; GWAS study and analysis IDs are summarized in Table 4. The pathway study was designed in two stages. The stage I contains *11* Framingham Heart Study (FHS) GWAS [37], and FHS traits include diabetes incidence, fasting plasma glucose (FPG), fasting Insulin (FI), insulin sensitivity, HbA1c and HOMA-IR. At stage I, pathway empirical p-value (*p*_*e*_) was calculated for every MSigDB gene set and summary of gene set’s association was computed as *Chi*2 = ∑_*i*_ − 2 ∗ *log*((*p*_*e*_)_*i*_) over all GWAS. Gene sets were ranked in decreasing order of Chi2 and top *500* gene sets (~5%) were selected for validation in the stage II of *5* independent GWAS (Table 4).

**Table 4:** **dbGaP GWAS of diabetes traits for common pathway study**

**Study name**	**Study ID**	**Analysis ID**	**Phenotype**	**N_SNPs**	**PMID**
**Stage I**					
FHS	phs000007	pha000417	Diabetes incidence	112923	17903298
FHS	phs000007	pha000423	Age-sex adjusted Fasting Plasma Glucose	112923	17903298
FHS	phs000008	pha000427	Age-sex adjusted Fasting Insulin	112923	17903298
FHS	phs000009	pha000429	Age-sex adjusted Insulin Sensitivity	112923	17903298
FHS	phs000010	pha000433	Age-sex adjusted HbA1c	112923	17903298
FHS	phs000011	pha000437	Age-sex adjusted HOMA-IR	112923	17903298
FHS	phs000012	pha000447	Multivariable adjusted Fasting Plasma Glucose	112923	17903298
FHS	phs000013	pha000451	Multivariable adjusted Fasting Insulin	112923	17903298
FHS	phs000014	pha000453	Multivariable adjusted Insulin Sensitivity	112923	17903298
FHS	phs000015	pha000457	Multivariable adjusted HbA1c	112923	17903298
FHS	phs000016	pha000461	Multivariable adjusted HOMA-IR	112923	17903298
**Stage II**					
FUSION	phs000100	pha002839	Type 2 Diabetes	306417	17463248
T1DGC	phs000180	pha002862	Type 1 Diabetes	503180	19430480
GoKinD	phs000018	pha002864	Diabetic Nephropathy	358475	21277817
SardiNIA	phs000338	pha003005	serum insulin	347043	
NFBC66	phs000276	pha002901	serum insulin	318890	19060910

Compared to low-resolution genotyping of FHS GWAS in stage I, GWAS of stage II contained approximately *300,000-500,000* SNPs with association p-values for pathway analysis, and the traits include T2D, T1D, diabetic nephropathy and serum insulin. Pathway adjusted p-value (*p*_*adj*_) was calculated for every validated gene set and a significant common pathway is defined as having *P*_*adj*_ ≤ 0.05 over all GWAS. Component genes of significant gene sets were examined and a significant common gene is identified if it has uniform score ≤ 0.05 over all GWAS.

## Additional files

Additional file 1: Table S2. Uniform scores of significant genes from identified common gene sets over all GWAS. Additional file 2: Table S3. Function characteristics of significant genes from identified common gene sets. Additional file 3: Table S1. MSigDB gene-set types.

## Abbreviations

USGSA: Uniform-score gene-set analysis
TFs: Transcription factors
GWAS: Genome-wide association studies
GSA: Gene-set analysis
HbA1c: Hemoglobin A1c
FPG: Fasting plasma glucose
T1D: Type I diabetes
T2D: Type 2 diabetes
MZ: Monozygotic
DZ: Dizygotic
ARIC: Atherosclerosis Risk in Communities
TSS: Transcription start site
TES: Transcription end site
pid: pathway id
KEGG: Kyoto Encyclopedia of Genes and Genomes
FHS: Framingham Heart Study
FI: Fasting Insulin
FWER: Family-wise error rate
CNS: Central nervous system
GPR: Group positive rate
