# Supplementary material for: The uniform-score gene set analysis for identifying common pathways associated with different diabetes traits
Source: BMC Genomics. 2015 Apr 23;16(1):336. doi: 10.1186/s12864-015-1515-3 (PMC4415316; doi:10.1186/s12864-015-1515-3)
Supplement: Additional file 2: Table S3. — Function characteristics of significant genes from identified common gene sets. [file 12864_2015_1515_MOESM2_ESM.docx]

**Table S3: function characteristics of significant genes from identified common gene sets**

| **Gene** | **OMIM** | **Entrez** | **Map** | **Tissue** | **Function** |
| --- | --- | --- | --- | --- | --- |
| NRXN1 | 600565 | 9378 | 2p16.3 | brain | schizophrenia[[1](#_ENREF_1)], autism[[2](#_ENREF_2)], intellectual disability[[3](#_ENREF_3)] |
| LRP1B | 608766 | 53353 | 2q21.2 | brain | tumor[[4](#_ENREF_4)] |
| CNTN4 | 607280 | 152330 | 3p26.3 | testis, lymph node, brain | development delay and 3p deletion syndrome[[5](#_ENREF_5)] |
| GRM7 | 604101 | 2917 | 3p26.1 | brain | hearing[[6](#_ENREF_6)] and schizophrenia[[7](#_ENREF_7)] |
| ROBO2 | 602431 | 6092 | 3p12.3 | brain, lung | kidney function[[8](#_ENREF_8)], neurodevelopment[[9](#_ENREF_9)] |
| CPNE4 | 604208 | 131034 | 3q22.1 | brain | neurodevelopment[[10](#_ENREF_10)] |
| PDE4D | 600129 | 5144 | 5q12 | skeletal muscle, kidney, lung, brain | brain function[[11](#_ENREF_11),[12](#_ENREF_12)] and stroke[[13](#_ENREF_13)] |
| SYNE1 | 608441 | 23345 | 6q25 | brain, ovary, kidney, … | brain function[[14](#_ENREF_14),[15](#_ENREF_15)] |
| ELMO1 | 606420 | 9844 | 7p14.1 | brain | diabetic nephropathy[[16](#_ENREF_16)] |
| MAGI2 | 606382 | 9863 | 7q21.11 | brain | brain function[[17](#_ENREF_17)], Usher syndrome[[18](#_ENREF_18)] |
| CNTNAP2 | 604569 | 26047 | 7q35 | brain | Autism[[19](#_ENREF_19)], language[[20](#_ENREF_20)] |
| SGCZ | 608113 | 137868 | 8p22 | brain | brain function[[21](#_ENREF_21)], obesity[[22](#_ENREF_22)], epilepsy[[23](#_ENREF_23)] |
| PTPRD | 601598 | 5789 | 9p24-p23 | brain | tumor[[24](#_ENREF_24)] |
| CTNNA3 | 607667 | 29119 | 10q21.3 | heart, brain | Alzheimer's disease[[25](#_ENREF_25)] |
| NELL1 | 602319 | 4745 | 11p15.1 | brain, kidney | neurodevelopment[[26](#_ENREF_26)], inflammatory bowel disease[[27](#_ENREF_27)] |
| DLG2 | 603583 | 1740 | 11q14.1 | brain | tumor[[28](#_ENREF_28)] |
| NTM | 607938 | 50863 | 11q25 | brain | neurodevelopment[[29](#_ENREF_29)] |
| PCDH9 | 603581 | 5101 | 13q21.32 | brain | autims[[30](#_ENREF_30)] |
| GPC6 | 604404 | 10082 | 13q32 | liver, colon, ovary, kidney | omodysplasia[[31](#_ENREF_31)] |
| NPAS3 | 609430 | 64067 | 14q13.1 | brain | schizophrenia, psychiatric illness[[32](#_ENREF_32)] |
| NRXN3 | 600567 | 9369 | 14q31 | brain | autism[[33](#_ENREF_33)] |
| RYR3 | 180903 | 20192 | 15q14 | brain | learning[[34](#_ENREF_34)] |
| RORA | 600825 | 6095 | 15q22.2 | skeletal muscle, blood, brain, … | autism[[35](#_ENREF_35)] |
| RBFOX1 | 605104 | 54715 | 16p13.3 | brain | brain function[[36](#_ENREF_36)] |
| ASIC2 | 601784 | 40 | 17q12 | brain | brain function[[37](#_ENREF_37)] |

OMIM: the access ID of gene description at NCBI Online Mendelian Inheritance in Man[[38](#_ENREF_38)]

Entrez: the gene ID of Entrez Gene database[[39](#_ENREF_39)]

Tissue: it describes the tissue where the gene is mainly expressed. The identification is based on expression annotation from AceView[[40](#_ENREF_40)].

Function: it listed main functions of the gene associated with diseases. The identification is based on citation analysis of Scopus[[41](#_ENREF_41)] database and review of gene annotations from Entrez[[39](#_ENREF_39)], OMIM[[38](#_ENREF_38)] and AceView[[40](#_ENREF_40)] gene annotation.

**References**:

1. Kirov G, Gumus D, Chen W, Norton N, Georgieva L, et al. (2008) Comparative genome hybridization suggests a role for NRXN1 and APBA2 in schizophrenia. Hum Mol Genet 17: 458-465.

2. Kim HG, Kishikawa S, Higgins AW, Seong IS, Donovan DJ, et al. (2008) Disruption of neurexin 1 associated with autism spectrum disorder. Am J Hum Genet 82: 199-207.

3. Dabell MP, Rosenfeld JA, Bader P, Escobar LF, El-Khechen D, et al. (2013) Investigation of NRXN1 deletions: clinical and molecular characterization. Am J Med Genet A 161A: 717-731.

4. Sonoda I, Imoto I, Inoue J, Shibata T, Shimada Y, et al. (2004) Frequent silencing of low density lipoprotein receptor-related protein 1B (LRP1B) expression by genetic and epigenetic mechanisms in esophageal squamous cell carcinoma. Cancer Res 64: 3741-3747.

5. Fernandez T, Morgan T, Davis N, Klin A, Morris A, et al. (2004) Disruption of contactin 4 (CNTN4) results in developmental delay and other features of 3p deletion syndrome. Am J Hum Genet 74: 1286-1293.

6. Friedman RA, Van Laer L, Huentelman MJ, Sheth SS, Van Eyken E, et al. (2009) GRM7 variants confer susceptibility to age-related hearing impairment. Hum Mol Genet 18: 785-796.

7. Ohtsuki T, Koga M, Ishiguro H, Horiuchi Y, Arai M, et al. (2008) A polymorphism of the metabotropic glutamate receptor mGluR7 (GRM7) gene is associated with schizophrenia. Schizophr Res 101: 9-16.

8. Grieshammer U, Le M, Plump AS, Wang F, Tessier-Lavigne M, et al. (2004) SLIT2-mediated ROBO2 signaling restricts kidney induction to a single site. Dev Cell 6: 709-717.

9. Hivert B, Liu Z, Chuang CY, Doherty P, Sundaresan V (2002) Robo1 and Robo2 are homophilic binding molecules that promote axonal growth. Mol Cell Neurosci 21: 534-545.

10. Prasad SS, Russell M, Nowakowska M, Williams A, Yauk C (2012) Gene expression analysis to identify molecular correlates of pre- and post-conditioning derived neuroprotection. J Mol Neurosci 47: 322-339.

11. Burgin AB, Magnusson OT, Singh J, Witte P, Staker BL, et al. (2010) Design of phosphodiesterase 4D (PDE4D) allosteric modulators for enhancing cognition with improved safety. Nat Biotechnol 28: 63-70.

12. Michot C, Le Goff C, Goldenberg A, Abhyankar A, Klein C, et al. (2012) Exome sequencing identifies PDE4D mutations as another cause of acrodysostosis. Am J Hum Genet 90: 740-745.

13. Gretarsdottir S, Thorleifsson G, Reynisdottir ST, Manolescu A, Jonsdottir S, et al. (2003) The gene encoding phosphodiesterase 4D confers risk of ischemic stroke. Nat Genet 35: 131-138.

14. Zhang Q, Skepper JN, Yang F, Davies JD, Hegyi L, et al. (2001) Nesprins: a novel family of spectrin-repeat-containing proteins that localize to the nuclear membrane in multiple tissues. J Cell Sci 114: 4485-4498.

15. Gros-Louis F, Dupre N, Dion P, Fox MA, Laurent S, et al. (2007) Mutations in SYNE1 lead to a newly discovered form of autosomal recessive cerebellar ataxia. Nat Genet 39: 80-85.

16. Shimazaki A, Kawamura Y, Kanazawa A, Sekine A, Saito S, et al. (2005) Genetic variations in the gene encoding ELMO1 are associated with susceptibility to diabetic nephropathy. Diabetes 54: 1171-1178.

17. Koide T, Banno M, Aleksic B, Yamashita S, Kikuchi T, et al. (2012) Common variants in MAGI2 gene are associated with increased risk for cognitive impairment in schizophrenic patients. PLoS One 7: e36836.

18. Bauss K, Knapp B, Jores P, Roepman R, Kremer H, et al. (2014) Phosphorylation of the Usher syndrome 1G protein SANS controls Magi2-mediated endocytosis. Hum Mol Genet 23: 3923-3942.

19. Alarcon M, Abrahams BS, Stone JL, Duvall JA, Perederiy JV, et al. (2008) Linkage, association, and gene-expression analyses identify CNTNAP2 as an autism-susceptibility gene. Am J Hum Genet 82: 150-159.

20. Abrahams BS, Tentler D, Perederiy JV, Oldham MC, Coppola G, et al. (2007) Genome-wide analyses of human perisylvian cerebral cortical patterning. Proc Natl Acad Sci U S A 104: 17849-17854.

21. Piovani G, Savio G, Traversa M, Pilotta A, De Petro G, et al. (2014) De novo 1Mb interstitial deletion of 8p22 in a patient with slight mental retardation and speech delay. Mol Cytogenet 7: 25.

22. Zhao W, Wineinger NE, Tiwari HK, Mosley TH, Broeckel U, et al. (2012) Copy number variations associated with obesity-related traits in African Americans: a joint analysis between GENOA and HyperGEN. Obesity (Silver Spring) 20: 2431-2437.

23. Baulac S, Gourfinkel-An I, Couarch P, Depienne C, Kaminska A, et al. (2008) A novel locus for generalized epilepsy with febrile seizures plus in French families. Arch Neurol 65: 943-951.

24. Stallings RL, Nair P, Maris JM, Catchpoole D, McDermott M, et al. (2006) High-resolution analysis of chromosomal breakpoints and genomic instability identifies PTPRD as a candidate tumor suppressor gene in neuroblastoma. Cancer Res 66: 3673-3680.

25. Miyashita A, Arai H, Asada T, Imagawa M, Matsubara E, et al. (2007) Genetic association of CTNNA3 with late-onset Alzheimer's disease in females. Hum Mol Genet 16: 2854-2869.

26. Liu M, Grigoriev A (2005) Fast parsers for Entrez Gene. Bioinformatics 21: 3189-3190.

27. Franke A, Hampe J, Rosenstiel P, Becker C, Wagner F, et al. (2007) Systematic association mapping identifies NELL1 as a novel IBD disease gene. PLoS One 2: e691.

28. Mazoyer S, Gayther SA, Nagai MA, Smith SA, Dunning A, et al. (1995) A gene (DLG2) located at 17q12-q21 encodes a new homologue of the Drosophila tumor suppressor dIg-A. Genomics 28: 25-31.

29. Struyk AF, Canoll PD, Wolfgang MJ, Rosen CL, D'Eustachio P, et al. (1995) Cloning of neurotrimin defines a new subfamily of differentially expressed neural cell adhesion molecules. J Neurosci 15: 2141-2156.

30. Marshall CR, Noor A, Vincent JB, Lionel AC, Feuk L, et al. (2008) Structural variation of chromosomes in autism spectrum disorder. Am J Hum Genet 82: 477-488.

31. Maglott D, Ostell J, Pruitt KD, Tatusova T (2005) Entrez Gene: gene-centered information at NCBI. Nucleic Acids Res 33: D54-58.

32. Pickard BS, Pieper AA, Porteous DJ, Blackwood DH, Muir WJ (2006) The NPAS3 gene--emerging evidence for a role in psychiatric illness. Ann Med 38: 439-448.

33. Vaags AK, Lionel AC, Sato D, Goodenberger M, Stein QP, et al. (2012) Rare deletions at the neurexin 3 locus in autism spectrum disorder. Am J Hum Genet 90: 133-141.

34. Balschun D, Wolfer DP, Bertocchini F, Barone V, Conti A, et al. (1999) Deletion of the ryanodine receptor type 3 (RyR3) impairs forms of synaptic plasticity and spatial learning. EMBO J 18: 5264-5273.

35. Nguyen A, Rauch TA, Pfeifer GP, Hu VW (2010) Global methylation profiling of lymphoblastoid cell lines reveals epigenetic contributions to autism spectrum disorders and a novel autism candidate gene, RORA, whose protein product is reduced in autistic brain. FASEB J 24: 3036-3051.

36. Gehman LT, Stoilov P, Maguire J, Damianov A, Lin CH, et al. (2011) The splicing regulator Rbfox1 (A2BP1) controls neuronal excitation in the mammalian brain. Nat Genet 43: 706-711.

37. Askwith CC, Wemmie JA, Price MP, Rokhlina T, Welsh MJ (2004) Acid-sensing ion channel 2 (ASIC2) modulates ASIC1 H+-activated currents in hippocampal neurons. J Biol Chem 279: 18296-18305.

38. Hamosh A, Scott AF, Amberger JS, Bocchini CA, McKusick VA (2005) Online Mendelian Inheritance in Man (OMIM), a knowledgebase of human genes and genetic disorders. Nucleic Acids Res 33: D514-517.

39. Maglott D, Ostell J, Pruitt KD, Tatusova T (2011) Entrez Gene: gene-centered information at NCBI. Nucleic Acids Res 39: D52-57.

40. Thierry-Mieg D, Thierry-Mieg J (2006) AceView: a comprehensive cDNA-supported gene and transcripts annotation. Genome Biol 7 Suppl 1: S12 11-14.

41. Burnham JF (2006) Scopus database: a review. Biomed Digit Libr 3: 1.
