# Supplementary material for: The uniform-score gene set analysis for identifying common pathways associated with different diabetes traits
Source: BMC Genomics. 2015 Apr 23;16(1):336. doi: 10.1186/s12864-015-1515-3 (PMC4415316; doi:10.1186/s12864-015-1515-3)
Supplement: Additional file 3: Table S1. — MSigDB gene-set types. [file 12864_2015_1515_MOESM3_ESM.docx]

**Table S1: MSigDB gene-set types**

| **setType** | **Symbol** | **Name** |
| --- | --- | --- |
| 0 | c0 | C0: all gene sets |
| 1 | c1 | C1: positional gene sets |
| 2 | c2 | C2: curated gene sets |
| 3 | c2_cgp | C2_CGP: chemical and genetic perturbations |
| 4 | c2_cp | C2_CP: Canonical pathways |
| 5 | c2_biocarta | C2_CP:BIOCARTA: BioCarta gene sets |
| 6 | c2_kegg | C2_CP:KEGG: KEGG gene sets |
| 7 | c2_reactome | C2_CP:REACTOME: Reactome gene sets |
| 8 | c3 | C3: motif gene sets |
| 9 | c3_mir | C3_MIR: microRNA targets |
| 10 | c3_tft | C3_TFT: transcription factor targets |
| 11 | c4 | C4: computational gene sets |
| 12 | c4_cgn | C4_CGN: cancer gene neighborhoods |
| 13 | c4_cm | C4_CM: cancer modules |
| 14 | c5 | C5: GO gene sets |
| 15 | c5_bp | C5_BP: GO biological process |
| 16 | c5_cc | C5_CC: GO cellular component |
| 17 | c5_mf | C5_MF: GO molecular function |
| 18 | c6 | C6: oncogenic signatures |
| 19 | c7 | C7: immunologic signatures |
